# Sample size requirements for pilot randomised controlled trials with continuous outcomes: a simulation study

**DOI:** 10.1186/1745-6215-14-S1-P46

**Published:** 2013-11-29

**Authors:** Marion Teare, Alexandra Hayman, Munya Dimairo, Neil Shephard, Amy Whitehead, Stephen Walters

**Affiliations:** 1 University of Sheffield, Sheffield, UK

## Aims

There is lack of consensus over what sample size should be used for external pilot trials to inform the design of definitive parallel group superiority randomised controlled trial (RCT). The aim of this research was to investigate the sample size required to precisely estimate the key parameter associated with a continuous outcome, the standard deviation (SD).

## Methods

We use a simulation approach to examine the sampling distribution of the SD for a Normally distributed outcome. We assessed the precision of the estimates by calculating the relative gain in precision through the width of the 95% confidence interval (CI) and the percentage gain in precision per increase in sample size of 5 in each group.

## Results

The bias in the pooled SD estimate is negligible once the total pilot sample is 60 or above and the relative gain in precision (for each ten subjects added to the pilot) drops to below 10% once the total sample size is 70. When planning the full RCT for a continuous outcome, estimates generated by pilot samples of 70 will have the required power with close to 50% confidence when the true standardised effect size is above 0.2. Adjusting the required sample sizes for the full RCT to deliver the required power with 80% confidence can result in excessively large full planned RCTs.

## Conclusions

We recommend that an external pilot study needs at least a total of 70 measured subjects (35 per group) to estimate the pooled SD for a continuous outcome.

